# A Comparison of Solubility, Stability, and Bioavailability between Astilbin and Neoastilbin Isolated from *Smilax glabra* Rhizoma

**DOI:** 10.3390/molecules25204728

**Published:** 2020-10-15

**Authors:** Dan Zheng, Yi-Ting Ruan, Zhong-Ping Yin, Qing-Feng Zhang

**Affiliations:** Jiangxi Key Laboratory of Natural Product and Functional Food, College of Food Science and Engineering, Jiangxi Agricultural University, Nanchang 330045, China; zhengdan0829@163.com (D.Z.); elain1117@163.com (Y.-T.R.); yinzp2008@sina.com (Z.-P.Y.)

**Keywords:** astilbin, neoastilbin, solubility, stability, bioavailability

## Abstract

Astilbin and neoastilbin are two flavonoid stereoisomers. In the present study, their solubility, stability, and bioavailability were compared in a rat. The results revealed that the water solubility of astilbin and neoastilbin was 132.72 μg/mL and 217.16 μg/mL, respectively. The oil–water distribution coefficient (log P) of astilbin and neoastilbin in simulated gastric fluid (SGF) was 1.57 and 1.39, and in simulated intestinal fluid (SIF) was 1.09 and 0.98, respectively. In SIF, about 78.6% astilbin remained after 4 h of incubation at 37 °C, while this value was 88.3% for neoastilbin. Most of the degraded astilbin and neoastilbin were isomerized into their cis-trans-isomer, namely neoisoastilbin and isoastilbin, respectively, and the decomposed parts were rare. For bioavailability comparison in a rat, an HPLC method for trace amounts of astilbin and neoastilbin determination in plasma was developed, and the pretreatment of plasma was optimized. A pharmacokinetic study showed that the absolute bioavailability of astilbin and neoastilbin in a rat showed no significant difference with values of 0.30% and 0.28%, respectively.

## 1. Introduction

Astilbin, (2*R*,3*R*)-3,3′,4′,5,7-pentahydroxyflavanon-3-α-l-rhamnopyranoside, is a dihydroflavonol found in many plants and plant-based foods, e.g., *Smilax glabra* Rhizoma (SGR) [1], *Engelhardtia roxburghiana* [2], grape, and wine [3]. According to the structure of astilbin, it has two asymmetric carbon atoms at C-2 and C-3 and, therefore, has four stereoisomers, namely neoastilbin (2*S*, 3*S*), astilbin (2*R*, 3*R*), neoisoastilbin (2*S*, 3*R*), and isoastilbin (2*R*, 3*S*) [4]. Generally, these four stereoisomers naturally coexist in plants. However, astilbin is usually the most dominant one, and there are many separation and purification methods to obtain high-purity astilbin, e.g., high-speed counter-current chromatography [5] and high-performance centrifugal chromatography [6]. Hence, its biological activity has also been extensively studied, including antioxidant activity, hypoglycemic effect [7], selective immunosuppressive activity [8], etc. Nevertheless, because of the low content of the other three stereoisomers in plants, there are few relevant studies on them.

The isomerism of compounds may significantly affect their bioactivity and/or physicochemical properties, and some drugs have completely different pharmacological effects between the two enantiomers. For instance, neoastilbin has a sweet taste, but astilbin does not [9]. Deoxynivalenol (DON) is a mycotoxin naturally found in cereal grains, with high toxicity to humans and livestock. However, its stereoisomer, 3-epi-DON, is substantially less toxic [10]. S-isomers of cefpodoxime proxetil has higher resistance to enzyme metabolism and lower degradation in the gastric region compared to R-isomers, which lead to significant bioavailability advantages [11].

**Scheme 1:**
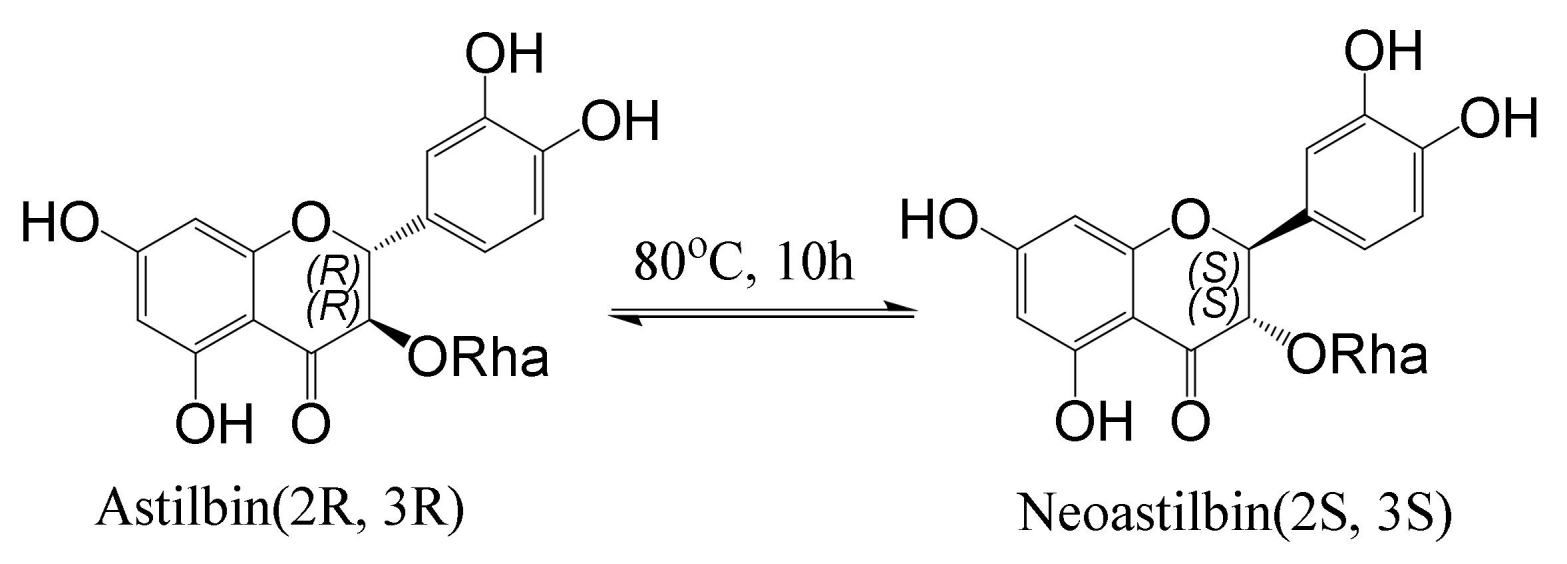
The transformation of astilbin to neoastilbin.

In our previous study, the interconversion between astilbin and its three stereoisomers was studied in detail. It was found that astilbin was mainly isomerized into neoastilbin with incubation at pH 7.0 for 10 h at 80 °C (Scheme 1). Hence, a novel and simple method for preparative separation of astilbin and neoastilbin from SGR was developed [12]. In the present study, the solubility, oil–water distribution coefficient (log P) and the stability of astilbin and neoastilbin were compared. Meanwhile, an HPLC method for quantification of trace amounts of astilbin and neoastilbin in plasma was developed, and their bioavailability in a rat was compared for the first time.

## 2. Results and Discussion

### 2.1. Solubility Comparison between Astilbin and Neoastilbin

**Figure 1:**
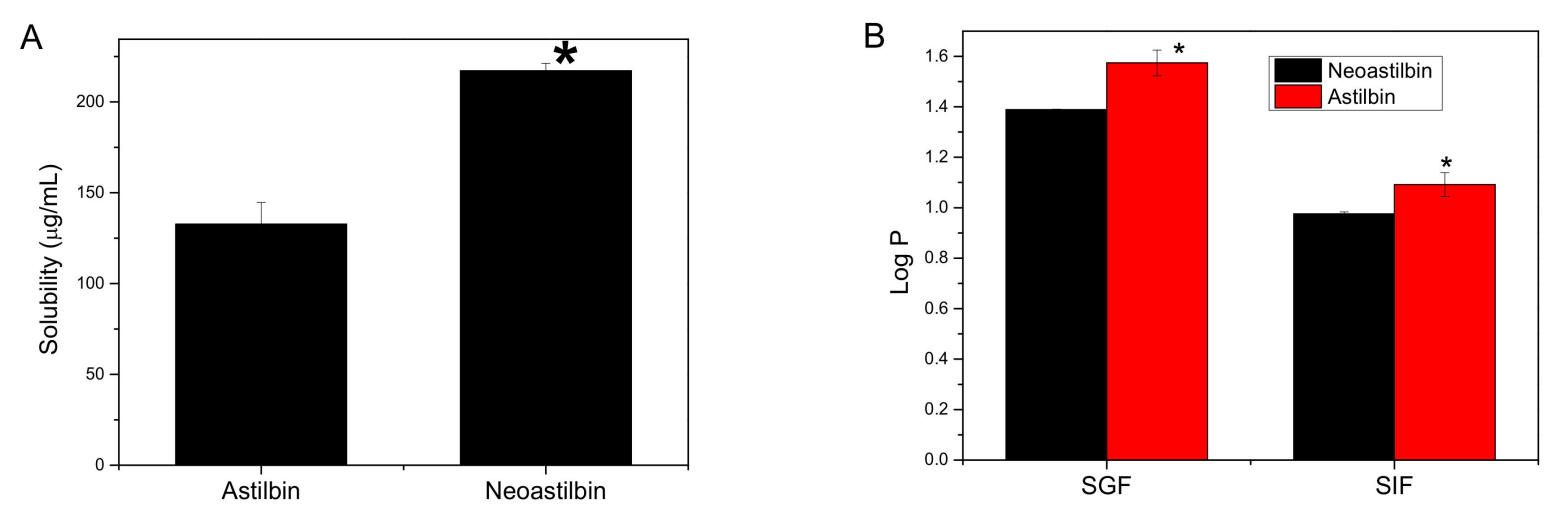
(**A**) The solubility of astilbin and neoastilbin in water at 25 °C (n = 3). (**B**) The oil–water distribution (log P) of astilbin and neoastilbin in the simulated gastric fluid (SGF) and simulated intestinal fluid (SIF) at 37 °C (n = 3). * means significant difference between astilbin and neoastilbin (*p* < 0.05).

The effect of temperature on the solubility of astilbin was previously studied [13]. The results showed that its solubility quickly increased with the rise of temperature. In the present study, the solubility of astilbin and neoastilbin was compared in water at 25 °C only. Figure 1A showed that the solubility of astilbin was 132.72 μg/mL, which was nearly half of neoastilbin (217.16 μg/mL). According to the criterion of Chinese Pharmacopoeia, with solubility in the range of 0.1–1 g/L at 25 °C [14], they are both very slightly soluble compounds. The solubility of astilbin was determined by the spectrophotometric method, with a value of 221 μg/mL in our previous study [13]. The bigger value was due to the isomerization of astilbin during the experiment. However, the isomers in the solution were all determined as astilbin through the spectrophotometric method. After realizing the isomerization in a subsequent study [12], the solubility of astilbin/neoastilbin was determined by HPLC in the present study, which can separate astilbin from its isomers. 

### 2.2. Oil–Water Distribution Coefficient (Log P) Comparison between Astilbin and Neoastilbin

The absorption of flavonoids in the small intestine mainly relies on passive diffusion because of a lacking receptor in the cell membrane [15]. Log P is a parameter that can partly reflect the ability of a compound to diffuse across phospholipids membrane [16]. When log P < 0, the drug is not easy to be absorbed in the intestinal tract and is only suitable for vascular administration. When log P > 3, the drug is too fat soluble to release from the cell membrane into nearby blood vessels and lymphatics. When 0 < log P < 3, the drug can be absorbed in the gastrointestinal tract; while 2 < log P < 3 is generally considered to be well absorbed [17]. Figure 1B shows that the log P of astilbin and neoastilbin were 1.57 and 1.39 in SGF, while these values were 1.09 and 0.98 in simulated intestinal fluid (SIF), respectively. The decrease of log P in SIF was attributed to the dissociated of hydroxyl group in flavonoids with the rise of pH, resulting in an increase of its hydrophilia. It was also noted that neoastilbin had smaller log P values than astilbin in both SIF and SGF, which indicated that the neoastilbin was more hydrophilic than astilbin. The results were consistent with the solubility test. The small values of log P of neoastilbin and astilbin in SIF may be due to the existence of rhamnose moiety in their structure. It is generally recognized that the lipophilicity of flavonoid glycoside is weaker than its aglycone [18]. The small intestine is the main region responsible for flavonoid absorption. These results indicate that neoastilbin and astilbin can be partly absorbed in the small intestine, but the bioavailability was very poor. 

### 2.3. Stability Comparison between Astilbin and Neoastilbin in SGF and SIF

**Figure 2:**
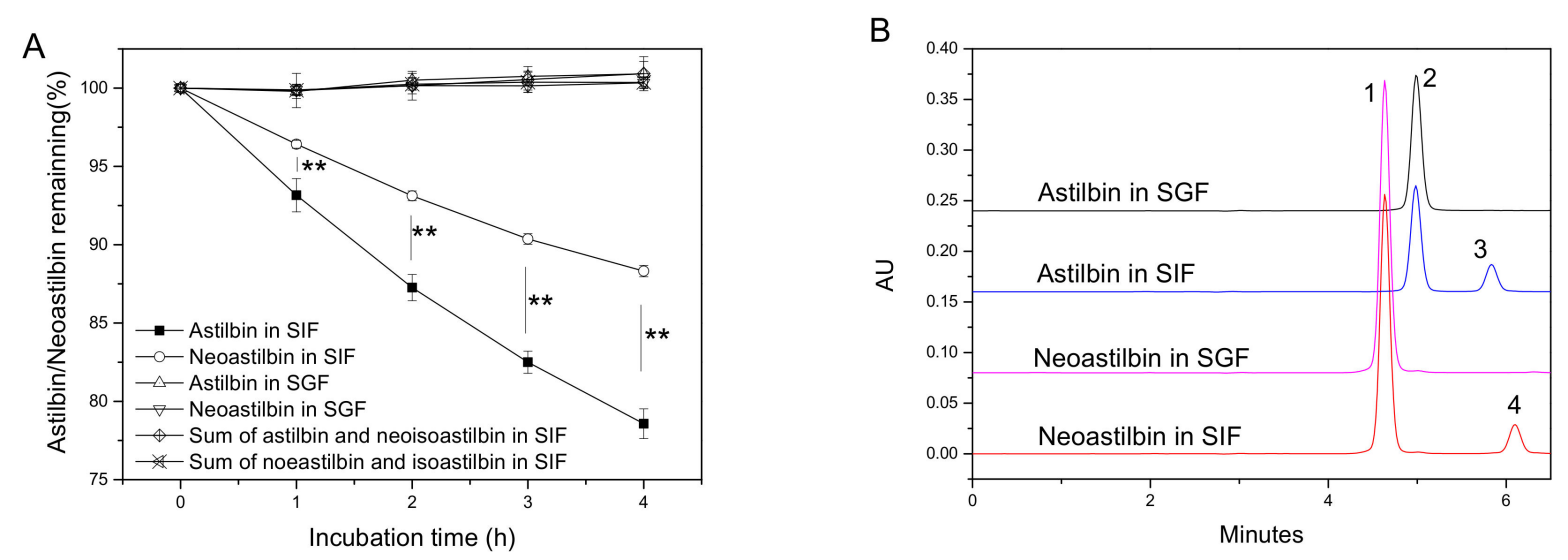
(**A**) Stability of astilbin and neoastilbin in SGF and SIF at 37 °C (n = 3). ** means very significant difference (*p* < 0.01) between remaining astilbin and neoastilbin under the same incubation time in SIF. (**B**) The chromatograms of neoastilbin and astilbin after incubation in SIF and SGF for 4 h at 37 °C, respectively. Peaks: 1, neoastilbin; 2, astilbin; 3, neoisoastilbin; 4, isoastilbin.

The stability of astilbin and neoastilbin in SGF and SIF was also compared. As shown in Figure 2A, astilbin and neoastilbin were very stable in SGF. Almost no decrease of their concentrations were found with 4 h of incubation in SGF. However, in SIF, the remaining astilbin and neoastilbin stably decreased with the incubation time, and astilbin decreased more rapidly. After 4 h of incubation, the remaining astilbin and neoastilbin was 78.6% and 88.3%, respectively. The results indicated that neoastilbin was more stable than astilbin. The interaction between the rhamnose and B ring moiety in the conformation of neoastilbin, as revealed by an NMR analysis, made it more stable [4]. Most of the degradated astilbin and neoastilbin were isomerized into their cis-trans-isomer, namely neoisoastilbin and isoastilbin, respectively (Figure 2B) [12]. The degradation of astilbin and its isomers was pH and temperature dependent. In neutral solution (pH 6–7), the decomposition rate was very slow, and isomerization was the dominant reaction. However, in alkaline solution (pH 8–10), the decomposition sped up rapidly with the rise of pH [19]. The peak area sum of astilbin and neoisoastilbin (or neoastilbin and isoastilbin) was almost unchanged with the incubation in SIF at pH 6.8 (Figure 2A). After oral administration, the degradation of flavonoids in the gastrointestinal tract may have notably affected their bioavailability. The results indicated that astilbin and neoisoastilbin were relatively stable in the gastrointestinal tract, and only isomerization, but not decomposition, was found. 

### 2.4. Plasma Sample Pretreatment Optimization for Pharmacokinetic Study

There are many plasma pretreatment methods for pharmacokinetic study, e.g., protein precipitation and liquid–liquid extraction [20]. An effective pretreatment could eliminate endogenous matrix components and reduce the consumption of preparation time. Two pretreatment methods—ethyl acetate liquid–liquid extraction and the protein precipitation method—were compared in the present study. The results showed that the absolute recovery of astilbin with ethyl acetate extraction was less than 60%, and the preparation time was too long. In the protein precipitation method, the effects of two precipitators, methanol and acetonitrile, were compared. It was found that acetonitrile caused the plasma protein aggregating immediately, and the recovery of astilbin (<50%) was far lower than that of methanol. Furthermore, its chromatographic peak was asymmetric and a serious leading peak was found (Figure S1A, Supplementary Materials). In comparison, when 150 μL methanol was used as the precipitator, the recovery of astilbin and neoastilbin was both bigger than 85% (Table S1, Supplementary Materials), and the chromatographic peak was symmetric and sharp (Figure S1B, Supplementary Materials). Thus, 150 μL of methanol was used to precipitate the protein in the plasma sample (50 μL) in this study. After centrifugation at 10,000× *g* for 10 min, about 190 μL supernatant was obtained. 

**Figure 3:**
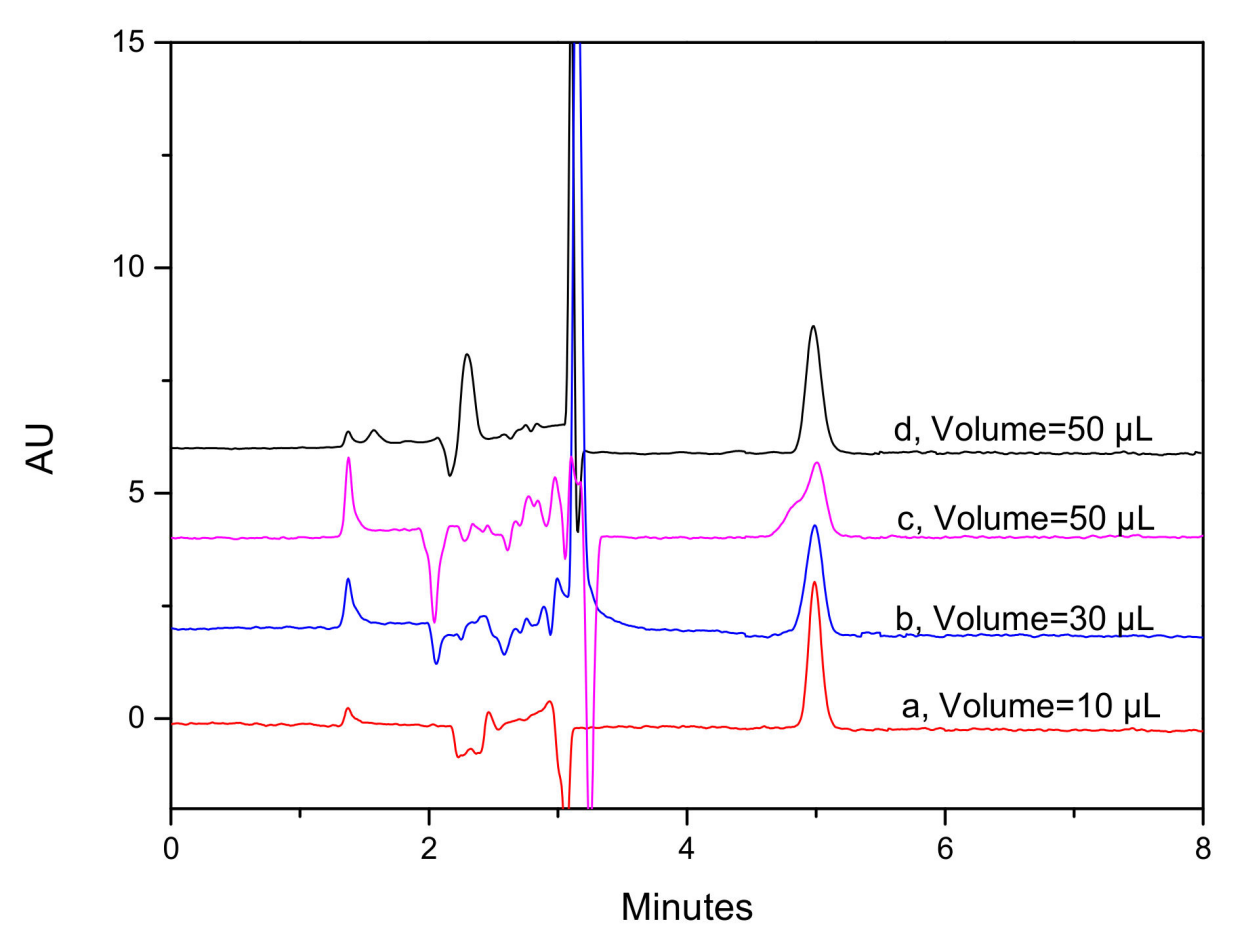
The chromatogram of astilbin in 75% methanol with different injection volumes (a, b, c) and in 40% methanol with 50 μL of injection volume (d).

**Figure 4:**
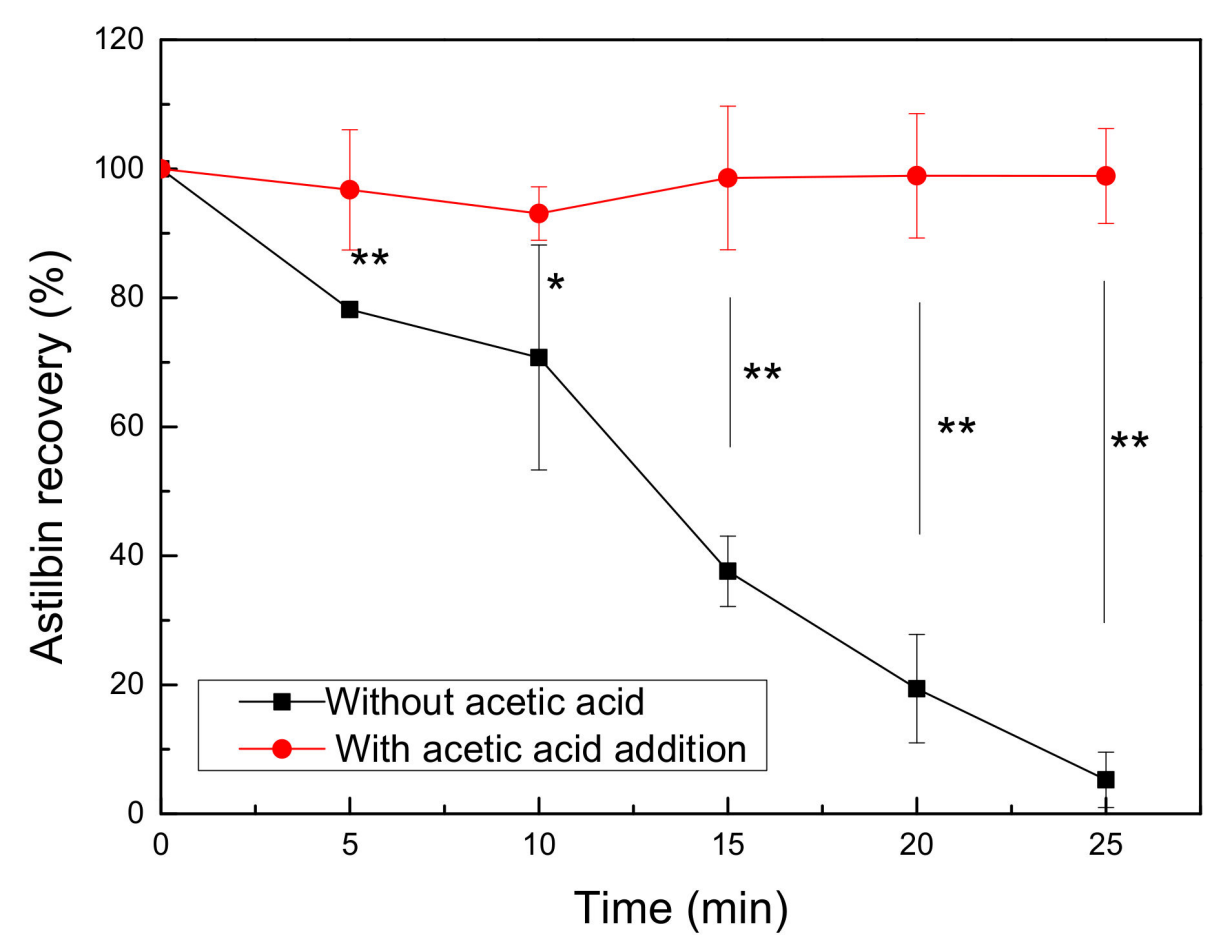
The stability of astilbin in extracting supernatant during concentration at 75 °C; * means significant difference (*p* < 0.05) and ** means very significant difference (*p* < 0.01) between the two treatments.

It is well know that the larger the sample injection volume, the more sensitive the HPLC method is. However, when the sample contains high amounts of organic solvent (e.g., methanol or acetonitrile), a large volume of sample injection may temporarily change the composition of the mobile phase, which may interfere with the shape and retention time of the chromatographic peak. In Figure 3, 10 μL, 30 μL, and 50 μL of 75% methanol, containing the same amount of astilbin, was injected for HPLC analysis. It clearly showed that bigger injection volume caused worse peak shape and peak front. However, when decreasing the methanol content in the sample (<40%), 50 μL of injection volume showed no obvious effect on peak shape. Hence, concentration to reduce the methanol content in the supernatant sample was necessary for large sample injection in present study. Methanol was evaporated at 75 °C in a water bath. Because the supernatant sample was alkalescent (~pH 9), the recovery of astilbin during concentration decreased rapidly (Figure 4), which was in accordance with the stability study that astilbin decomposes quickly in alkaline solution at high temperatures [19]. Hence, acetic acid was used to acidize the sample solution and further stabilize astilbin. When 5 μL acetic acid was added, the recovery of astilbin was almost 100% during concentration (Figure 4). Similar results were found for neoastilbin. Concentration-time with 10–12 min was chosen in the present study. During the time, 190 μL of sample solution was concentrated to about 90 μL in a 1.5 mL centrifuge tube. After centrifugation at 10,000× *g* for 10 min again, the concentrate was used for HPLC analysis with injection volume of 50 μL.

The stability of astilbin/neoastilbin in a plasma sample was also evaluated in detail. The results are summarized in Table 1. With storage at room temperature for 2–8 h (−25 °C), the recovery of astilbin and neoastilbin in plasma was within 89.23–106.16%, and no obvious degradation was found. Similar results were also found in long-time storage of plasma at −80 °C. With three freeze/thaw cycles, the recovery of astilbin and neoastilbin was 97.15 and 93.29%, respectively. These results showed that astilbin and neoastilbin were relatively stable in plasma, and that routine analysis duration would not cause the degradation of them. 

### 2.5. HPLC Method Development for Pharmacokinetic Study

Figure S2 (Supplementary Materials) showed the chromatograms of blank plasma and spiked sample with astilbin, neoastilbin, and internal standard (IS). As shown, the retention times of astilbin, neoastilbin, and IS under the chromatographic condition were 4.97, 4.62, and 3.47 min, respectively. No interferential peak was found in the blank sample.

Table 2 lists the regression equation, precision, and accuracy of the HPLC quantitative method. The calibration curves of astilbin and neoastilbin were linear over the concentration range of 17.3–1105 ng/mL and 31.6–1010 ng/mL, respectively. The lower limit of quantitative limit (LLOQ) and lower limit of detection limit (LLOD) of the method were calculated by the calibration curve with signal/noise ratio (S/N) of 10 and 3, respectively. The results showed that the LLOD and LLOQ for astilbin were 4.8 ng/mL and 16.3 ng/mL, and for neoastilbin, 5.3 ng/mL and 17.5 ng/mL, respectively. In comparison, Guo et al. developed an HPLC method for astilbin determination in rabbit plasma, and the LLOQ was 0.44 μM (198 ng/mL) [21]. 

The precision and accuracy of the method was determined by using a spiked plasma sample with 100 ng/mL of astilbin/neoastilbin. The intra-day and inter-day accuracies of astilbin were 94.56% and 92.75%, while these values were 96.15% and 91.27% for neoastilbin, respectively. The intra-day and inter-day precision of migration times were 0.34% and 0.21% for astilbin, while these values were 0.35% and 0.23% for neoastilbin. The intra-day and inter-day precision of the peak area values were 1.49% and 3.71% for astilbin, while these values were 7.78% and 4.23% for neoastilbin. 

The validation results indicated that the developed HPLC method was sensitive and stable for pharmacokinetic study of astilbin and neoastilbin in rat plasma.

### 2.6. Bioavailability Comparison between Astilbin and Neoastilbin in Rat

**Figure 5:**
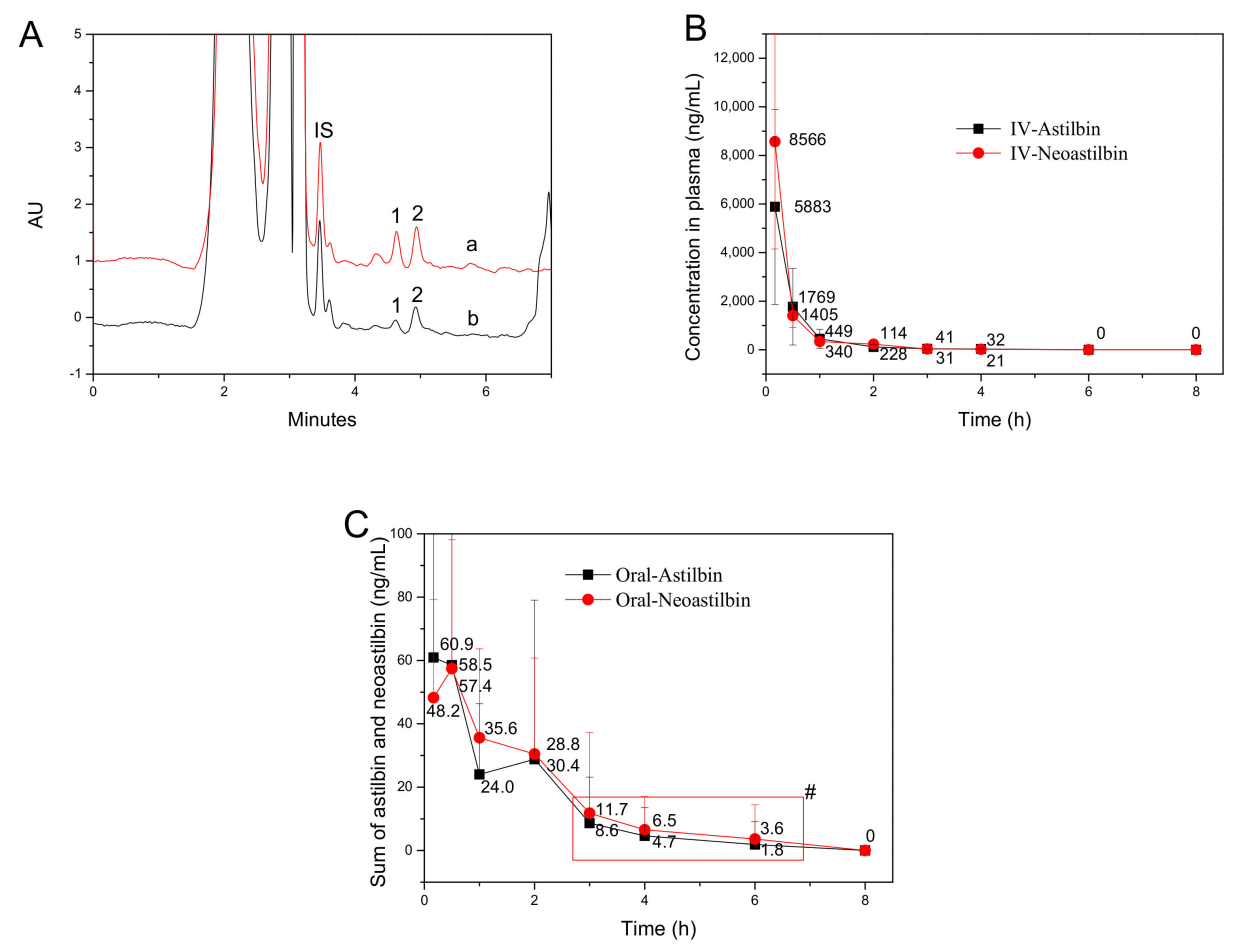
(**A**) Typical chromatograms of rat plasma at 30 min after oral administration of astilbin (a) and neoastilbin (b) (detected at 291 nm); (**B**) mean plasma concentration–time curves of astilbin and neoastilbin after intravenous administration (2 mg/kg) in rat. (**C**) Mean plasma concentration–time curves of astilbin and neoastilbin after oral administration of astilbin and neoastilbin suspensions (20 mg/kg) in rat; peaks: 1, neoastilbin; 2, astilbin; IS, rutin. #These data in the red box were below the lower limit of quantitation (LLOQ) of the quantified method. Hence, the accuracy of these digits was uncertain, but the presence of the flavonoid in plasma was confirmed.

Figure 5A was the typical chromatograms of rat plasma at 30 min after oral administration of astilbin and neoastilbin, respectively. As shown, the peaks of neoastilbin (peak 1) and astilbin (peak 2) were clearly found in the chromatograms. It was also noted that the two isomers were found simultaneously in some plasma samples, although only one flavonoid was administrated to the rat. The results indicated that the isomerization between the two flavonoids occurred in the small intestine. Thus, the bioavailability was calculated using the area sum of the two peaks. The plasma concentration–time profiles of the two flavonoids with different administration routes are shown in Figure 5B,C, while the pharmacokinetic parameters simulated by software are summarized in Table 3.

After intravenous (IV) administration with a dose of 2 mg/kg of body weight, the plasma concentration of astilbin and neoastilbin decreased quickly with time. The maximal serum concentration (C_max_) values found at 0.17 h were 5883.4 ± 2081.0 and 8566.7 ± 3091.8 ng/mL for astilbin and neoastilbin, respectively. The other pharmacokinetic parameters, such as the time in which C_max_ is reached (T_max_), clearance (CL), and mean residence time (MRT)_(0-t)_ of the two flavonoids were very similar. However, the t_1/2_ was calculated to be 1.2 h for neoastilbin, which was longer than that of astilbin (0.5 h). After oral administration with a dose of 20 mg/kg of body weight, the C_max_ of astilbin was found at 0.17 h with a value of 60.9 ng/mL, while the C_max_ of neoastilbin was found at 0.5 h with a value of 57.5 ng/mL. Excluding these, no other significantly different parameters were found between the two isomers. The absolute bioavailability (Fr%) of astilbin and neoastilbin were 0.30% and 0.28%, respectively. The absorption of the two isomers in the rat was very poor and showed no significant difference. The poor bioavailability results are in accordance with the solubility and log P test. Astilbin exhibited novel immunosuppressive activity and is an attractive immunomodulator candidate [9]. Its three other isomers also showed similar immunosuppressive activity. In the present study, we found that astilbin and neoastilbin have no absorption difference, and the isomerization between them occurred in vivo. Hence, astilbin and its isomers may be considered as the same substance in drug development and it is unnecessary to purify a single component. 

Since there is no existence of a specific receptor for phenolic phytochemicals in intestinal epithelial cells, the absorption of these compounds is mainly based on passive diffusion in vivo [15]. Due to low solubility and permeability, astilbin and neoastilbin were poorly absorbed in the rat. Wang showed that the oral bioavailability of astilbin in the rat was only 0.066% [22]. However, its bioavailability in the rat, determined by Lei et al., was 2.01% [23]. In the present study, the oral bioavailability of astilbin and neoastilbin in the rat were 0.30% and 0.28%, respectively. All of these studies confirmed the poor bioavailability of astilbin. On the other hand, the absorbed flavonoids in vivo usually undergo the metabolism of deglycosylation, sulfation, glucuronide, and/or methylation in intestinal epithelial cells and hepatocytes. However, in the present study, the metabolites of astilbin and neoastilbin were neither identified nor quantified as yet. Further studies to improve the bioavailability of astilbin and its metabolite identification are in progress in our lab. 

## 3. Materials and Methods

### 3.1. Chemicals and Reagents

Astilbin (>98%) and neoastilbin (>97%) were purified from SGR in our laboratory, and were identified by UV, IR, MS, and NMR [12]. HPLC-grade acetonitrile and methanol were purchased from Anhui Tedia High Purity Solvents Co., Ltd. (Anqing city, Anhui province, China). (+)-Rutin trihydrate was purchased from Aladdin Industrial Co., Ltd. (Shanghai, China). Milli-Q water was used throughout the study. All other reagents used were analytical grade.

### 3.2. Solubility Test

An excessive amount of astilbin/neoastilbin (about 5 mg) was mixed with 2 mL of water in a tube with stopper. The mixture was shaken for 3 days at 25 °C in a water bath vibrator (HZQ-2, Changzhou, China). After filtering through 0.22 μm filter, the dissolved astilbin/neoastilbin was determined by HPLC.

### 3.3. Oil–Water Distribution Coefficient Determination

The oil–water distribution coefficient (log P) of astilbin/neoastilbin was determined by the “shake-flask” method. The partition equilibrium of the compound in two immiscible phases (n-octanol and water) is attained by shaking for 48 h, and then its concentration in each phase is measured. The method is advanced in its simple operation, low cost, and accuracy [24]. Briefly, simulated gastric fluid (SGF, pH 1.2) and simulated intestinal fluid (SIF, pH 6.8) without enzyme were prepared in accordance with the United States Pharmacopeia [25]. The *n*-octanol and SGF/SIF were first saturated together by shaking them for 48 h. After separation, the layer of SGF/SIF was used to dissolve astilbin/neoastilbin. Then, the astilbin/neoastilbin saturated SGF/SIF was mixed with an equal volume of n-octanol, and the mixture was shaken for 48 h to reach the equilibrium at 25 °C in an oscillator. The astilbin/neoastilbin concentrations in the layer of SGF/SIF (C_1_) and *n*-octanol (C_2_) were determined by HPLC. The log P was calculated with the following equation:

(1) $$\mathrm{LogP}=\log(\frac{C_{2}}{C_{1}})$$

### 3.4. Stability Comparison of Astilbin and Neoastilbin in SGF (pH 1.2) and SIF (pH 6.8)

Astilbin/neoastilbin was dissolved in 40% ethanol with a concentration of 1 mg/mL. A 1.0 mL aliquot of the astilbin/neoastilbin solution was mixed with 9 mL of SGF or SIF (without enzyme), respectively. Then, the mixture was incubated at 37 °C. At different time points (1, 2, 3, 4 h), the remaining astilbin/neoastilbin was determined by HPLC.

### 3.5. Bioavailability Comparison of Astilbin and Neoastilbin in Rats

Sprague–Dawley (SD) rats were purchased from the Hunan Slack Jingda Laboratory Animal Company, Ltd. (Changsha City, Hunan Province, China). Rats were bred with free access to water and food in an animal house with a 12:12 h light/dark cycle and temperature of 23–25 °C. The animal studies complied with guidelines of Jiangxi Agricultural University on animal care (Ethics Committee approval number: JXAULL-2020-29). 

Twenty-four female SD rats (300 ± 15 g) were randomly divided into four groups (n = 6). The rats fasted for 12 h with access to water prior to the administration. Two group rats were received IV administration of 0.3 mL of astilbin or neoastilbin solution (0.2 mg/mL) through the caudal vein with a dose of 2 mg/kg of body weight, respectively. The IV solution was prepared by diluting 50 times astilbin/neoastilbin stock solution (10 mg/mL dissolved in 50% ethanol) with water. The other two group rats were orally administered with 1 mL of astilbin or neoastilbin (6 mg/mL suspended in water) with a dose of 20 mg/kg of body weight, respectively. At 0.167, 0.5, 1, 2, 3, 4, 6, and 8 h after administration, blood samples (~200 μL) were collected from the tail vein into a K2-ethylenediaminetetraacetic acid (EDTA)-pretreated tube. After centrifugation at 2000× *g* for 10 min, the plasma samples were transferred to a new 1.5 mL tube and stored at −80 °C until analysis.

Pharmacokinetic parameters, including the maximal serum concentration (C_max_), the time in which C_max_ is reached (t_max_), the area under the concentration–time curve (AUC), the mean residence time (MRT), the clearance (CL), and the half-life in the terminal phase (t_1/2_), were simulated by Drug and Statistics (DAS) software (version 2.0) using a non-compartmental statistical model. The model does not require any compartmental modeling assumptions, which is more convenient in the pharmacokinetic experiment. Please refer to the review article by Barron et al. for details of this model [26]. The absolute bioavailability (Fr,%) was calculated as follows:

(2) $$\mathrm{Fr}(\%)=\frac{{\mathrm{AUC}}_{\mathrm{oral}}}{{\mathrm{AUC}}_{\mathrm{iv}}}\times 100$$

### 3.6. Plasma Sample Pretreatment 

#### 3.6.1. Preparation of Spiked Plasma Sample

An aliquot of 10 μL astilbin/neoastilbin standard solution (~100 μg/mL in 40% ethanol) was mixed with 1 mL of blank plasma to obtain the spiked plasma sample (~1 μg/mL).

#### 3.6.2. Plasma Sample Pretreatment Method Comparison 

The extraction efficiency of astilbin/neoastilbin from the spiked plasma sample by ethyl acetate liquid–liquid extraction and the protein precipitation method was compared. 

Ethyl acetate liquid–liquid extraction: an aliquot of 50 μL spiked plasma sample was extracted by 100 μL of ethyl acetate for three times by vortexing. After centrifugation, the ethyl acetate layer was obtained and combined together. After drying by nitrogen, the residue was re-dissolved by 100 μL 50% ethanol. After centrifugation at 10,000× *g* for 10 min, the supernatant was used for HPLC analysis. The absolute recovery of astilbin was calculated as follows:

(3) $$\mathrm{Recovery}(\%)=\frac{\mathrm{Extracted}\;\mathrm{astilbin}\;}{\mathrm{Absolute}\;\mathrm{astilbin}\;\mathrm{added}\;\mathrm{in}\;\mathrm{plasma}}\times 100$$

Protein precipitation method: methanol and acetonitrile used as protein precipitators were compared. Briefly, an aliquot of 50 μL spiked plasma sample was mixed with a different volume of methanol or acetonitrile (150, 200, 250 μL), respectively. After vortexing for 2 min, the mixture was centrifugated at 10,000× *g* for 10 min. The supernatant was used for HPLC analysis and the absolute recovery was calculated.

#### 3.6.3. Astilbin/Neoastilbin Stability in Plasma Sample

The stability of astilbin/neoastilbin in rat plasma was investigated by analyzing the recovery of astilbin/neoastilbin in spiked plasma sample (~1 μg/mL) under different storage conditions in triplicates. For short-time stability, the spiked sample was stored at an ambient atmosphere for various time (2 h, 4 h, 6 h, and 8 h). For long-time stability, the spiked sample was stored at −80 °C for four weeks, and the recovery of astilbin/neoastilbin was determined each week. The freeze/thaw stability was performed after three freeze/thaw cycles (−80 °C to room temperature).

#### 3.6.4. Plasma Sample Pretreatment for Astilbin/Neoastilbin Stability and Pharmacokinetic Study

Briefly, an aliquot of 50 μL plasma sample was mixed with 150 μL of methanol containing 350 ng/mL rutin (used as internal standard, IS), and 5 μL of acetic acid was added subsequently. The mixture was vortexing for 2 min. After centrifugation at 10,000× *g* for 10 min, the supernatant was transferred into a new centrifuge tube and then concentrated at 75 °C for 12 min in a water bath. The concentrate was centrifuged at 10,000× *g* for 10 min again, and the supernatant was used for HPLC analysis. When the sample concentration was higher than the upper limit of the calibration range, appropriate dilution was made.

### 3.7. HPLC Method Development for Pharmacokinetic Study

#### 3.7.1. HPLC Conditions

The analysis was performed on an Agilent 1260 HPLC system (Agilent Technologies, Palo Alto, CA) equipped with an autosampler and diode array detector. A symmetry^®^ C18 column (4.6 mm × 250 mm, 5.0 μm; Waters, USA) was used. Mobile phase consisted of 25% acetonitrile and 75% water (with 0.1% acetic acid). The flow rate was 1.0 mL/min with column temperature of 40 °C and detection wavelength of 291 nm (for astilbin and neoastilbin) and 355 nm (for rutin). The injection volume was 50 μL and the analysis duration was 8 min.

#### 3.7.2. Calibration Curve

The astilbin/neoastilbin spiked plasma sample (~1 μg/mL) was diluted with blank plasma to obtain a series of concentrations for calibration development. The calibration curve was constructed by plotting the peak area ratio of astilbin (or neoastilbin)/rutin (Y) against astilbin (or neoastilbin) concentration (X).

#### 3.7.3. Precision and Accuracy

Intra-day precision and accuracy were assessed in a single day by determining the spiked plasma sample (100 ng/mL) in six replicates. Similarly, inter-day precision and accuracy were evaluated on three consecutive days. The measured concentration of the spiked plasma samples (100 ng/mL) was calculated by using the calibration curve and compared with the nominal concentration.

### 3.8. Statistical Analysis

Data were expressed as mean ± standard deviation of triplicates. All data analysis was performed with software of Origin 8.5 (Origin Lab Co., Northampton, MA, USA).

## 4. Conclusions

The physicochemical properties of astilbin and neoastilbin were compared. They are both very slightly soluble compounds, and neoastilbin shows relatively better solubility. The small values of log P of neoastilbin and astilbin in SIF indicated that they can, with difficultly, pass through the cell membrane of the intestinal epithelial cell. Neoastilbin and astilbin underwent isomerization in SIF, and almost no decomposition was found. A plasma pretreatment procedure and an HPLC method were successfully developed for the pharmacokinetic study. Bioavailability comparison results showed that the absorption of neoastilbin and astilbin in the rat was very poor and showed no significant difference.

## Figures and Tables

**Table 1:** Stability of astilbin and neoastilbin in plasma (1 μg/mL) under different store conditions. (n = 3).

Conditions	Stability	Relative Recovery (%) ^1^	
		Astilbin	Neoastilbin
**Short-time** **(Ambient Atmosphere)**	2 h	106.16 ± 2.76	92.38 ± 1.04 *
	4 h	98.02 ± 3.48	89.38 ± 1.73 *
	6 h	101.10 ± 5.71	89.23 ± 4.48 *
	8 h	95.60 ± 5.28	96.30 ± 6.03
**Long-time** **(−80 °C)**	7 days	86.34 ± 2.17	87.78 ± 8.01
	14 days	91.52 ± 2.48	92.40 ± 4.46
	21 days	89.04 ± 8.91	89.57 ± 9.25
	28 days	86.65 ± 3.35	86.87 ± 6.60
**Freeze/Thaw**	Three freeze/thaw	97.15 ± 7.45	93.29 ± 10.32

^1^ The astilbin/neoastilbin concentration in plasma was calculated through the calibration curve in Table 2. * means significant difference (*p* < 0.05) between astilbin and neoastilbin.

**Table 2:** The accuracy, precision (Relative standard deviation, RSD), linearity, lower limit of detection (LLOD), and lower limit of quantitation (LLOQ) of astilbin and neoastilbin in rat plasma (~100 ng/mL). (n = 3).

Compound	Migration Time (RSD,%)		Peak Area Ratio (RSD,%)		Accuracy (%)		Regression Equation	Linear Range (ng/mL)	Linearity (R)	LLOD (ng/mL)	LLOQ (ng/mL)
	Intra-Day	Inter-Day	Intra-Day	Inter-Day	Intra-Day	Inter-Day					
Astilbin	0.34	0.21	1.49	3.71	94.56	92.75	Y = 2.324X	17.3–1105	0.9915	4.8	16.3
Neoastilbin	0.35	0.23	7.78	4.23	96.15	91.27	Y = 2.189X	31.6–1010	0.9975	5.3	17.5

**Table 3:** The main pharmacokinetic parameters after intravenous (IV) administration (2 mg/kg) and oral administration (PO) of astilbin and neoastilbin (20 mg/kg) in rat. (n = 6).

	IV-Ast	IV-Neoast	PO-Ast	PO-Neoast
C_max_ (ng/L)	5883.4 ± 2081.0	8566.7 ± 3091.8	60.9 ± 48.1	57.5 ± 25.3
AUC_(0-t)_ (ng/L·h)	3487.1 ± 1813.2	4370.9 ± 1417.1	103.8 ± 56.8	118.3 ± 73.5
AUC_(0-∞)_ (ng/L·h)	3493.4 ± 1793.2	4480.3 ± 1420.0	107.5 ± 50.4	122.1 ± 88.2
T_max_ (h)	0.17 ± 0	0.17 ± 0	0.17 ± 0.5	0.5 ± 0.4
t_1/2_ (h)	0.5 ± 0.5	1.2 ± 0.1	1.36 ± 0.7	1.2 ± 0.7
CL (L·h^−1^·kg^−1^)	0.5 ± 0.4	0.5 ± 0.1	186.0 ± 22.8	163.7 ± 17.9
MRT_(0-t)_	0.4 ± 0.2	0.4 ± 0.1	1.5 ± 0.2	1.67 ± 0.3
MRT_(0-∞)_	0.4 ± 0.3	0.5 ± 0.1	1.7 ± 1.4	2.0 ± 0.7
Fr(%)	100	100	0.30	0.28

## Supplementary Materials

The following are available online: Figure S1. The chromatogram of astilbin spiked plasma sample (1 μg/mL) treated with different volumes of methanol (**A**) and acetonitrile (**B**); Figure S2. The chromatogram of astilbin, neoastilbin, and IS (rutin). (**A**) blank rat plasma sample; (**B**) blank rat plasma sample spiked with astilbin (detected at 291 nm); (**C**) blank rat plasma sample spiked with neoastilbin (detected at 291 nm); (**D**) blank rat plasma sample spiked with IS (detected at 355 nm). Table S1. The recovery of astilbin and neoastilbin in spiked plasma (~1 μg/mL) treated with different volumes of methanol (n = 3).
