# Supplementary material for: A Comparison of Solubility, Stability, and Bioavailability between Astilbin and Neoastilbin Isolated from Smilax glabra Rhizoma
Source: Molecules. 2020 Oct 15;25(20):4728. doi: 10.3390/molecules25204728 (PMC7587598; doi:10.3390/molecules25204728)
Supplement: Supplementary file 1 [file molecules-25-04728-s001.pdf]

molecules

Supplementary Materials

A Comparison of Solubility, Stability, and Bioavailability between Astilbin and Neoastilbin Isolated from *Smilax glabra* Rhizoma

Dan Zheng, Yi-Ting Ruan, Zhong-Ping Yin and Qing-Feng Zhang *

Jiangxi Key Laboratory of Natural Product and Functional Food, College of Food Science and Engineering, Jiangxi Agricultural University, Nanchang 330045, China; zhengdan0829@163.com (D.Z.); elain1117@163.com (Y.-T.R.); yinzp2008@sina.com (Z.-P.Y.)

* Correspondence: zhqf619@126.com; zhqf619@jxau.edu.cn; Tel.: +86-791-3813863 (Q.F.)

Received: 30 August 2020; Accepted: 12 October 2020; Published: date

Academic Editor: Nawaf Al-Maharik

Abstract: Astilbin and neoastilbin are two flavonoid stereoisomers. In the present study, their solubility, stability, and bioavailability were compared in a rat. The results revealed that the water solubility of astilbin and neoastilbin was 132.72 μg/mL and 217.16 μg/mL, respectively. The oil–water distribution coefficient (log P) of astilbin and neoastilbin in simulated gastric fluid (SGF) was 1.57 and 1.39, and in simulated intestinal fluid (SIF) was 1.09 and 0.98, respectively. In SIF, about 78.6% astilbin remained after 4 h of incubation at 37 °C, while this value was 88.3% for neoastilbin. Most of the degraded astilbin and neoastilbin were isomerized into their cis-trans-isomer, namely neoisoastilbin and isoastilbin, respectively, and the decomposed parts were rare. For bioavailability comparison in a rat, an HPLC method for trace amounts of astilbin and neoastilbin determination in plasma was developed, and the pretreatment of plasma was optimized. A pharmacokinetic study showed that the absolute bioavailability of astilbin and neoastilbin in a rat showed no significant difference with values of 0.30% and 0.28%, respectively.

Keywords: astilbin; neoastilbin; solubility; stability; bioavailability

Table S1. The recovery of astilbin and neoastilbin in spiked plasma (1 μg/mL) treated with different volumes of methanol (n = 3).

Volume(μL)	Recovery (%)	
	Astilbin	Neoastilbin
150	85.35±3.86	86.49±2.70
200	82.47±1.09	84.18±0.74
250	79.09±1.50	84.75±3.07

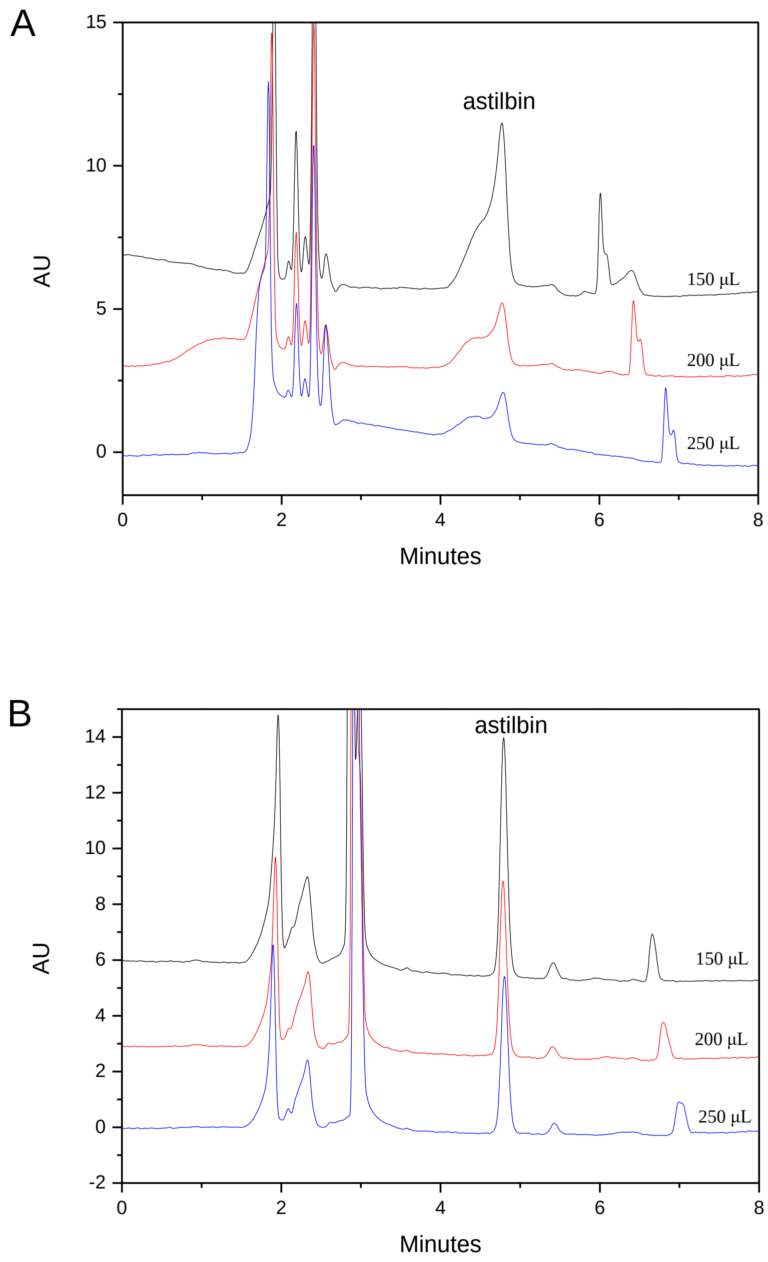

Figure S1. The chromatogram of astilbin spiked plasma sample (1 μg/mL) treated with different volumes of acetonitrile (A) and methanol (B).

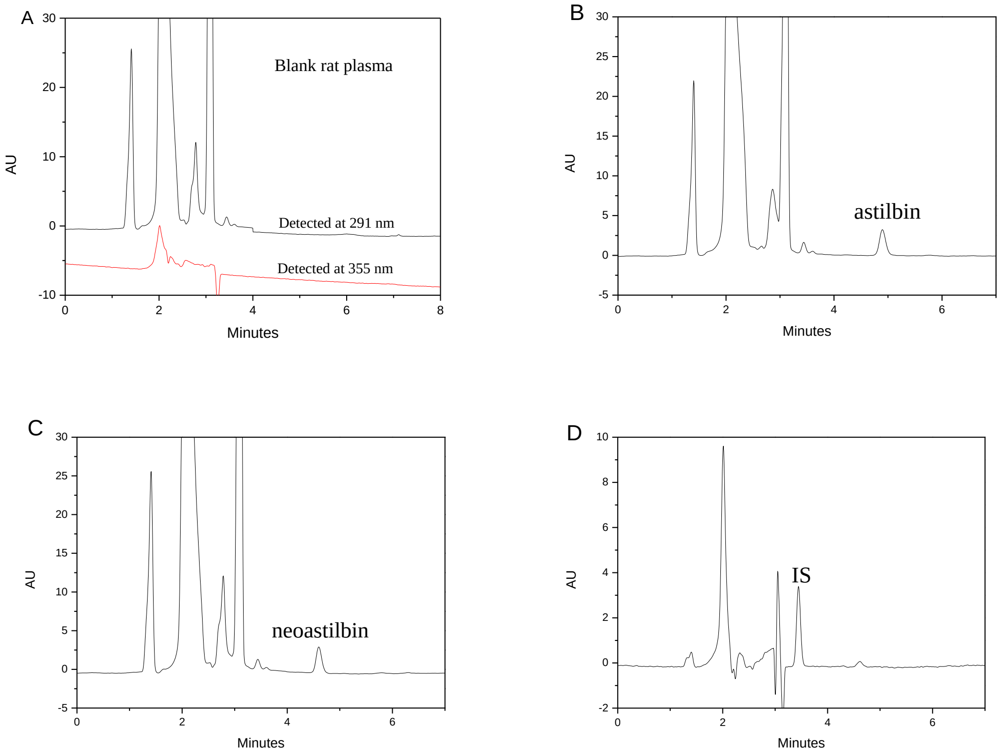

Figure S2. The chromatograms of astilbin, neoastilbin and IS (rutin). (A) blank rat plasma sample; (B) blank rat plasma sample spiked with astilbin (detected at 291 nm); (C) blank rat plasma sample spiked with neoastilbin (detected at 291 nm); ; (D) blank rat plasma sample spiked with IS (detected at 355 nm) .

Sample Availability: Samples of the compounds of astilbin and neoastilbin are available from the authors.

© 2020 by the authors. Submitted for possible open access publication under the terms and conditions of the Creative Commons Attribution (CC BY) license (http://creativecommons.org/licenses/by/4.0/).
